# Institutionalizing family care responsibility in China’s multilevel medical security system: evidence from long-term care insurance policy texts through a Confucian care ethics lens

**DOI:** 10.3389/fpubh.2026.1918294

**Published:** 2026-07-31

**Authors:** Chuanzhao Chen, Yigang Zhang, Peng Liu

**Affiliations:** 1 School of Politics and Public Administration, Qufu Normal University, Rizhao, China; 2 Law School, Qufu Normal University, Rizhao, China; 3 Academy for China's Rule-of-Law, East China University of Political Science and Law, Shanghai, China

**Keywords:** Confucian care ethics, family care responsibility, health policy, long-term care insurance, multilevel medical security, policy text analysis, public health governance

## Abstract

**Background:**

China’s long-term care insurance reform is a key component of multilevel medical security for addressing care needs among older adults with substantial functional decline, and it also raises an ethical governance question: how family-based Confucian care responsibilities are translated into modern rules of eligibility, payment, service provision and regulation.

**Methods:**

This study analyzed core long-term care insurance policy texts from 48 pilot cities through directed content analysis, a 45-item coding framework, policy text indices, K-means clustering and a supplementary comparison with the 2026 national rollout document. The 48-city sample was drawn from the 49-city national pilot framework after one city was excluded because no eligible core implementation text was located.

**Results:**

Local policy texts most clearly institutionalized needs assessment, payment responsibility and socialized service provision. Home-based or visiting care was widely recognized, but more specific caregiver-support rules, including caregiver training, respite care, family participation in care planning and detailed home-care service packages, remained less developed, as did digital governance. The national rollout document strengthened equity protection and family care responsibility, but detailed caregiver support and age-friendly digital access still require further specification.

**Conclusion:**

Long-term care insurance in China functions not only as a financing and service instrument within multilevel medical security, but also as a public health governance mechanism that translates filial responsibility, care for vulnerable older adults, people-centered governance and benevolent rule into institutional arrangements.

## 1 Introduction

The World Health Organization defines long-term care as the continuing arrangement of support that helps people with significant declines in physical or mental capacity maintain functional ability and dignity (1, 2). This definition moves older-age care beyond the boundaries of private family life and places it within the agenda of public health governance. In China, care for older adults has long been embedded in family ethics and intergenerational responsibility. Filial piety emphasizes the duty of family members to care for older parents, and recent studies show that filial norms shape care choices and understandings of family responsibility among older adults with substantial functional decline (3, 4). Yet long-term care needs are enduring, professional, and financially demanding. Individual families increasingly struggle to carry the full burden of care. The 2016 and 2020 national pilot documents that introduced and expanded long-term care insurance therefore represent an institutional response to this pressure (5, 6).

Long-term care insurance has developed as a medical security practice within this real pressure and cultural context. In this study, multilevel medical security refers to China’s layered public protection system in which basic medical insurance, medical assistance, supplementary protection mechanisms, and related care-security arrangements jointly address health and care risks. Existing research has examined the consequences of long-term care insurance for health improvement (7), expenditure sharing and service utilization (8–10), family care and subjective wellbeing (11, 12), and caregiver labor supply (13), showing that the system has entered the fields of public health governance, family welfare, and care-resource allocation (14, 15). Yet observing policy implementation only through health or economic outcomes cannot explain how local policy texts define care risks associated with functional decline during institutional construction, allocate payment responsibility within the multilevel medical security system, organize service provision, connect digital governance, and incorporate family care responsibility. Policy text evidence is therefore needed to examine the institutional rules through which care responsibility is written before implementation outcomes can be observed. This study uses policy text content analysis to examine how core policy texts from 48 pilot cities express care responsibility, service provision, and governance mechanisms, and uses the 2026 national rollout document as a supplementary textual reference to compare how long-term care insurance translates Confucian cultural meanings within modern medical security and public health governance in China.

## 2 Literature review

### 2.1 Long-term care insurance and public health governance

Research on long-term care insurance has shown that once care needs among older adults with substantial functional decline enter a public protection system, observable consequences appear in health, expenditure, and service use. Health studies place long-term care insurance within the governance of health risks among middle-aged and older adults and suggest that institutional intervention may improve health status (7). Studies on financial burden extend this analysis to household economic pressure by examining whether long-term care insurance can buffer out-of-pocket expenditure associated with care needs (16). Comparative studies of benefit design and pilot schemes further show that different payment methods, benefit rules, and pilot models are associated with differences in medical utilization, expenditure outcomes, and subjective wellbeing (8–12). This body of work moves from health improvement to expenditure sharing, service use, and benefit design, indicating that the public health value of long-term care insurance comes not only from care benefits themselves, but also from the institutional sharing of long-term care risks.

A second line of policy-effect research extends long-term care insurance from individual health and financial burden to family life and care organization. Studies on family welfare show that long-term care insurance changes the welfare conditions under which older adults and their families face care risks (11, 12). Research on formal and informal care further shows that when institutionalized care services enter family life, they reshape family care arrangements and may affect the health of spouses (14, 17). Studies on caregiver labor supply extend policy consequences to the allocation of work and care time among family members (13, 18). Chinese studies have also examined the effects of long-term care insurance on health, medical expenditure, family care, ageing-in-place patterns, and institutional care supply (19–21). Together, these studies move long-term care insurance from a single insurance benefit to a problem of public health governance, family welfare, and care resource allocation.

These studies demonstrate the multidimensional consequences of long-term care insurance, but their main explanatory focus remains on changes after policy implementation. Health improvement, expenditure sharing, service use, and family behavioral changes show what policy effects have occurred, but they say less about whether these effects rest on clear textual foundations during the stage of institutional construction. The ways local policy texts define care risks, allocate payment responsibility, organize service provision, and incorporate family care responsibility therefore require analysis beyond policy-effect studies (9). Policy text expression is not a repetition of outcome research. It is an entry point for understanding the responsibility-allocation logic behind observed policy consequences.

### 2.2 From family care to shared care responsibility

Family care research explains why care needs associated with substantial functional decline cannot remain entirely within private households. Care for older adults in China has long relied on family members, and caregiving pressure affects whether family care can be sustained (22). Family care resources and caregiver willingness shape the stability of daily support (23, 24), while social support and coping strategies influence the experience and consequences of caregiving burden (25). These studies reveal the practical boundary of family care: the family remains an important organizational unit for long-term care, but a single household cannot continuously bear the full risk of substantial care needs.

When formal services and public support enter family care settings, family responsibility is reorganized within a broader service network. Welfare-state theory helps clarify this reorganization by locating care and welfare risks within arrangements that involve the state, market, and family (26), while familialism research shows that public policy may assume, support, or ease the caring function of families (27). Applied to long-term care insurance, responsibility socialization describes the institutional process through which public rules recognize family care, provide support tools, and connect families with formal service networks. Integrated medical and older population care policies may change the pressure faced by family caregivers (28), and evidence from Shanghai indicates that formal care use can alter care input by adult children (15). Research on applicants for long-term care insurance further finds that type of functional decline, family structure, and care networks produce differences in the use of formal and informal care (29). Studies on social support systems for family care also emphasize that families caring for older adults with substantial functional decline or dementia need training, service referral, financial support, and community resources (30). These findings indicate that responsibility socialization keeps families within care relations while placing family care under public rules, service support, and institutional coordination.

Family care research explains why families need public institutional support and how formal care changes family arrangements, but its analysis often remains at the level of family experience and service use. Less is known about how local long-term care insurance policies write family responsibility into institutional rules, or how family care is converted into arrangements that can be paid for, served, and regulated. Family care research therefore provides not only background for this study, but also an empirical premise for shifting policy text analysis toward care responsibility allocation.

### 2.3 Confucian care ethics and public responsibility

Confucian culture provides a normative source for understanding long-term care responsibility in China, but this source does not fix care responsibility exclusively within the private family. The Classic of Filial Piety treats filial piety as the root of moral cultivation (31). Mencius extends care from one’s own older adults to the older population of others through the logic of moral extension (32). The Book of Rites imagines an ethical order in which older adults have secure later life and widowed, orphaned, childless, and disabled people are cared for (33). Mencius also provides a people-centered political ethic by placing the people above the state and ruler (32). Modern Confucian long-term care studies continue this concern. Confucian filial piety remains an important value resource for understanding responsibility for older population care, but long-term care must address the relationship among family responsibility, forms of care, and public support (34, 35). Social long-term care policy in Chinese culture cannot simply reject filial piety, nor can it push the entire care burden back to families. It must combine family, community, and formal services (36). Filial responsibility, care for vulnerable older adults, benevolent rule, and people-centered governance are therefore not parallel labels. They form a continuous normative chain from family care responsibility, to the response to the vulnerability of older adults with care needs, to the responsibility of public authority for livelihood risks.

Modern empirical studies further show that this normative chain enters long-term care practice only through institutional arrangements. Conceptual analysis of filial piety indicates that parental care includes duty, reciprocity, emotion, and the maintenance of family relationships (3). Research on filial culture and care choices also shows that cultural norms influence care preferences and understandings of family responsibility among older adults with substantial functional decline (4). In concrete family care settings, authoritarian filial piety, self-efficacy, and caregiver gains are linked in complex ways (23), while market transition and changing intergenerational support affect the psychological conditions of older parents (37). These studies suggest that filial tradition can explain the normative foundation of family care responsibility, but it cannot by itself explain how modern long-term care systems allocate public responsibility.

Research on state-family relations moves cultural norms into the field of social policy. Modern social policy redistributes risks between family responsibility and public responsibility, and Guan’s analysis of state-family relations in Chinese social policy provides a theoretical background for understanding how public protection systems intervene in family risks (38). Local social work research shows that filial culture can be reinterpreted in modern service practice (39). Medical and nursing studies also show that cultural factors affect family decision-making, communication, and acceptance of care (40), and research on family health management under Confucian cultural conditions suggests the need for institutional coordination between traditional ethics and modern medical services (41). These studies move Confucian culture from family ethics toward public service and institutional coordination.

Confucian culture is therefore used in this study as a lens for interpreting the normative meaning of policy texts, not as a direct object of measurement. Long-term care insurance policies usually express care responsibility through institutional language such as eligibility, payment responsibility, service provision, care-needs assessment, and family participation. They do not necessarily use intellectual-historical terms such as filial piety, benevolent rule, or people-centered governance. The central question is how local policies use modern medical security institutions to assume filial care, care for vulnerable older adults, and livelihood-related care responsibility, and how they translate family ethical responsibility into the language of public protection and care governance.

Existing studies have shown that long-term care insurance has public health consequences and have revealed the complex relationships among family care pressure, formal care entering family life, and Confucian cultural norms. What still needs explanation is how local pilot policy texts define care risks during institutional construction, allocate payment, service, and regulatory responsibilities among families, service providers, communities, and the state, and thereby provide modern institutional expression for filial responsibility, care for vulnerable older adults, benevolent rule, and people-centered governance. As long-term care insurance moves into national rollout, local pilot texts also become references for national institutional formation. This study therefore takes the core policy texts of 48 pilot cities as its main object of analysis and uses the national rollout document as a supplementary reference to examine what local institutional expressions are continued, strengthened, or left incomplete in the national text.

## 3 Methods

Local long-term care insurance policy texts contain institutional arrangements on covered populations, financing and payment, care-needs assessment, service provision, and regulation. To assess how these texts express care responsibility, this study first establishes stable sample boundaries and coding rules, and then converts textual elements into comparable indices and text types. The study uses directed content analysis, policy text indices, and cluster analysis to examine how local pilot policies allocate care responsibility among families, service providers, communities, and the state during institutional construction. It also uses the 2026 national rollout document as a supplementary reference to observe continuation, strengthening, and remaining gaps in national policy expression.

### 3.1 Study objects and sample inclusion

The sample frame is the national long-term care insurance pilot cities. The first national pilot document in 2016 launched long-term care insurance pilots, and the 2020 expansion document broadened the pilot scope (5, 6). This study collected core local policy texts according to the framework of 49 national pilot cities and included 48 cities after excluding one city without an eligible core implementation text. Inclusion was limited to implementation plans, implementation measures, opinions, detailed rules, and institutional management measures that specified covered populations, financing and payment, service provision, and management rules.

To ensure that the analysis corresponded to local institutional rules, the study excluded local news, policy introductions, single payment notices, service-site information, and explanatory webpages without institutional rules. Local policy texts were obtained from government websites, medical security or human resources and social security departments, local government gazettes, and manually verified legal databases. After textual verification, 48 cities entered the main analysis. Shihezi was not included because only a contribution collection plan for long-term care insurance was located and no core implementation text meeting the inclusion criteria was obtained.

### 3.2 Coding framework sources and operationalization

This study uses directed content analysis to convert institutional elements in local policy texts into observable coding items. Content analysis is suitable for turning policy text arrangements concerning objects, tools, responsibilities, and procedures into verifiable evidence (42–44). The first-level dimensions of the coding framework draw mainly on three sources. First, the 2016 and 2020 national pilot documents set institutional requirements for covered populations, financing and payment, care-needs assessment, service provision, regulation, and information systems. Second, existing policy evaluation studies of long-term care insurance show that local policies differ comparably in financing, covered populations, service provision, regulatory rules, and policy-tool combinations (9, 45). Third, long-term care and family care research indicates that home-based care, family participation, caregiver training, formal service provision, and public payment arrangements are important institutional links for understanding the reallocation of care responsibility (14, 15, 29, 30).

In this study, care responsibility allocation denotes the way policy texts assign, support, and regulate care-related duties among families, public medical security agencies, service providers, communities, and the state. The concept is operationalized through rules on eligibility, assessment, payment, service provision, family participation, caregiver support, digital access, and regulation. General moral statements about family duty or public responsibility are treated as background unless they are translated into specific institutional rules. On this basis, the study sets six first-level dimensions. The equity protection dimension captures how policies define covered groups and the ways vulnerable groups enter the system. The care-needs assessment and regulation dimension records eligibility determination, assessment procedures, review and appeal, and service regulation. The socialization of family care responsibility dimension refers to the institutional recognition, support, and reorganization of family care through public rules and services; it records whether home-based or visiting services extend the care setting into the family, whether family members are identified as participants in care relations, and whether family caregivers receive concrete support tools. The payment protection dimension records financing sources, fund management, payment standards, and benefit boundaries. The digital governance dimension records information platforms, medical security settlement, data sharing, electronic records, intelligent regulation, and data security. The socialized service provision dimension captures institutions, communities, designated service providers, service catalogs, care personnel, and integration between medical and old population care. Confucian care ethics guides the interpretation of these coded institutional elements; it does not function as an additional terminology code for filial piety, benevolent rule, or people-centered governance. The core meanings and typical second-level coding items are shown in Table 1, and the full coding-item sources, judgment rules, positive examples, and non-codable situations are provided in Supplementary Table S1.

**Table 1:** Coding framework and typical coding items.

First-level dimension	Core meaning	Second-level coding items
Equity protection	Covered populations and protection of vulnerable groups	Resident or broad population coverage; Support for low-income groups; Rural or primary-level accessibility; Priority for severe disability or dementia; Support for older adults living alone, empty-nest older adults, or very old adults; Prevention of digital exclusion; Informed consent and rights protection
Care-needs assessment and regulation	Eligibility confirmation and service constraint	Unified disability assessment standards; Third-party assessment; Clear assessment procedures; Reassessment and dynamic adjustment; Objection and review mechanism; Service provider regulation; Penalties for violations
Socialization of family care responsibility	Home-based care and support for family caregivers	Availability of home-based or door-to-door LTC services; Identification of family members or relatives as care actors; Training support for family members or relatives; Cash subsidies or benefit payments for family or relative care; Respite care; Family participation in assessment, care planning, or service choice; Home-based or door-to-door service package
Payment protection	Financing, fund management, and benefit payment	Separate accounting or fund management; Fiscal subsidies; Individual contributions; Employer or pooled-fund financing; Payment for home-based care; Payment for institutional care; Clear payment ratio; Clear payment ceiling; Clear benefit scope
Digital governance	Information construction, supervision, and data protection	LTCI information platform; Linkage with medical insurance settlement; Online application and assessment; Data sharing; Electronic care records; Intelligent review and regulation; Data security and privacy; Age-friendly digital services
Socialized service provision	Professional service system and service linkage	Availability of institutional care; Availability of community-based care; Designated service provider system; Diversified service providers; Clear service catalogue; Care worker qualifications; Integration of medical care and older population care

Full second-level coding sources, judgment rules, and non-codable situations are provided in Supplementary Table S1.

Each second-level item was coded as 1 when the policy text explicitly included the corresponding institutional arrangement and as 0 otherwise. LTCI, long-term care insurance. The six first-level dimensions are interpreted as institutional rule categories rather than moral vocabulary.

Coding followed a binary judgment rule. When a policy text explicitly included a corresponding arrangement on covered populations, financing and payment, service provision, assessment procedure, regulatory mechanism, or family support, the item was coded as 1. When the text did not include the corresponding institutional arrangement, or when it appeared only as a general principle such as reducing family burden, strengthening service management, or improving service quality without specific objects, procedures, payment rules, or service rules, the item was coded as 0. If such expressions appeared together with institutional arrangements such as home-based nursing, visiting services, family-member participation, family confirmation, family caregiver training, or caregiver subsidies, coding followed the specific rule for the corresponding item. In the family-care dimension, family participation required explicit roles in assessment, care-plan formulation, service choice, service confirmation, or informed signature; proxy application or contact-person information alone was insufficient. Family confirmation required rules allowing or requiring relatives to sign service records, confirm care delivery, or verify service use. Caregiver training required training, nursing guidance, or care-capacity building for family members or relative caregivers; training solely for professional care workers, assessors, or institutional staff was not coded. Local news, policy introductions, single payment notices, and explanatory webpages without institutional rules were not used as coding evidence. For ambiguous expressions, the study adopted a conservative coding principle and did not infer specific institutional elements from general policy goals.

### 3.3 Index construction and cluster analysis

The study first coded each second-level item as 0 or 1 and then calculated six dimensional indices at the city level. Each dimensional index equals the number of positively coded items in that dimension divided by the number of codable items in the same dimension, with values ranging from 0 to 1. A higher value indicates that a city policy text writes more institutional elements into that dimension.

Composite index methodology usually discusses index construction through indicator selection, weighting, aggregation, and interpretive boundaries. Related studies emphasize that an indicator system should serve a clear analytical object (46), that weights and aggregation methods should match the research purpose (47), and that composite index results should not be overinterpreted as a single quality ranking (48). In this study, second-level coding items are distributed under six first-level dimensions, and the number of items differs across dimensions. If all second-level items were directly summed, dimensions with more items, such as payment protection, would gain greater influence in the total score, allowing item count to substitute for the importance of the institutional dimension itself. To reduce this structural bias, the study first calculates six dimensional indices and then takes their equal-weighted average to form the comprehensive policy text index. Equal weighting functions as a transparent analytical convention for this textual analysis. The six dimensions represent distinct institutional functions through which policy texts express care responsibility allocation: equity protection, assessment and regulation, family-care responsibility socialization, payment protection, digital governance, and socialized service provision. In the absence of an external normative or empirical benchmark for assigning different weights to these first-level dimensions, equal-weight averaging prevents item-count differences from becoming implicit conceptual weights. The index is interpreted only as the relative expression strength of institutional elements in local policy texts. It is not interpreted as a ranking of local policy quality, implementation quality, or policy effects.

On the basis of descriptive statistics, the study uses K-means clustering to identify expression types in local pilot policy texts. The clustering variables are the six dimensional indices. The clustering results are used to observe combined differences in responsibility allocation, payment protection, service provision, and governance mechanisms across local policy texts (49–51). Cluster analysis in this study functions as an exploratory classification tool for summarizing policy text expression types and does not test policy effects. The three-cluster solution was retained because it provided an interpretable distinction among low-, moderate-, and high-expression policy text types and corresponded to the observed differences in family support, equity protection, digital governance, and service linkage.

### 3.4 Supplementary reference analysis of the national rollout document

Local pilot policy texts are not only materials of local institutional exploration. They also provide a reference for how the national long-term care insurance system compares with pilot-stage texts in the national rollout stage. The 2026 national rollout document (52) was not used to construct the coding framework or local city-level indices. It was coded only afterward as a supplementary comparison text under the same coding framework to compare common and different elements between the national rollout text and local pilot texts. This treatment provides textual evidence for discussing institutional correspondence, policy strengthening, and remaining gaps during the national rollout stage, and it further explains how long-term care insurance translates normative resources into covered populations, payment responsibility, service provision, and regulatory rules within modern public health governance. The comparison identifies textual alignment and gaps within the publicly available document, without supporting claims about policy diffusion, implementation quality, or legal validity.

## 4 Results

### 4.1 Six-dimensional structure of local pilot policy texts

**Figure 1:**
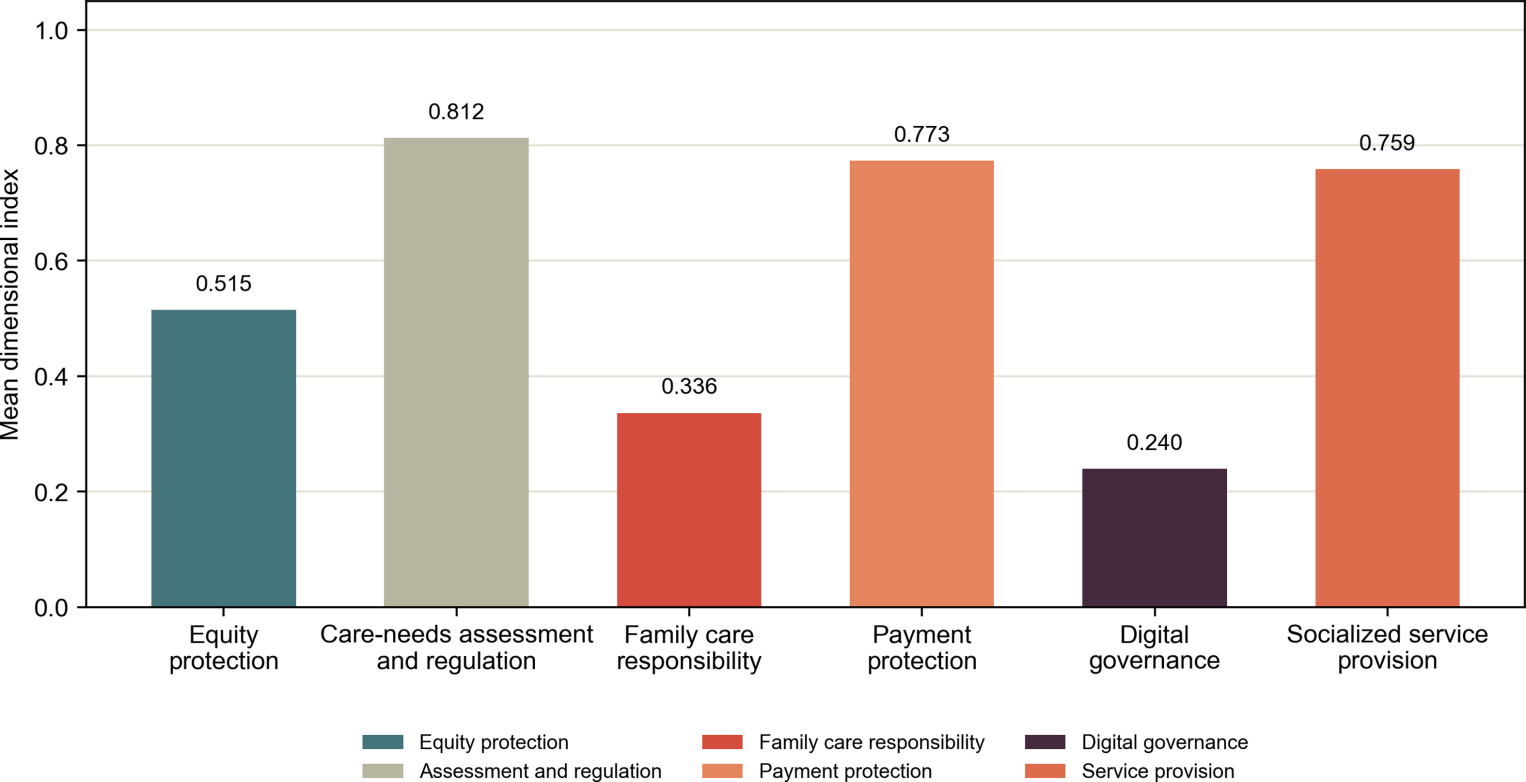
Mean values of the six policy text indices across the 48 pilot cities.

The 48-city policy texts show an uneven six-dimensional structure. Table 2 and Figure 1 reports the six dimensional indices. Care-needs assessment and regulation, payment protection, and socialized service provision are the most stable dimensions, with mean values of 0.812, 0.773, and 0.759, respectively. Equity protection has a mean value of 0.515. Socialization of family care responsibility and digital governance have mean values of 0.336 and 0.240, respectively. This pattern indicates that local pilot texts have more fully written assessment, payment, and service provision into institutional rules, whereas caregiver-support tools and digital accessibility remain less systematically expressed.

**Table 2:** First-level dimensional indices of policy texts in 48 cities.

First-level dimension	Mean	Median	Minimum	Maximum	Comparable cities
Equity protection	0.515	0.571	0.143	0.857	48
Care-needs assessment and regulation	0.812	0.857	0.429	1.000	48
Socialization of family care responsibility	0.336	0.286	0.000	0.857	48
Payment protection	0.773	0.778	0.222	1.000	48
Digital governance	0.240	0.250	0.000	0.750	48
Socialized service provision	0.759	0.714	0.429	1.000	48

Each index ranges from 0 to 1. Higher values indicate stronger textual expression of the corresponding dimension across the 48 cities.

### 4.2 Second-level coding-item frequencies and family support expression

Second-level coding-item frequencies further clarify where the stable and weak dimensions come from. Table 3 reports the most frequent second-level items. Assessment procedures and provider supervision appear in all 48 city texts, and clear payment proportion appears in 47 cities. Institutional care accessibility and diverse provider types appear in 45 cities, and medical-care integration appears in 44 cities. These high-frequency items concentrate in care-needs assessment and regulation, payment protection, and socialized service provision, which is consistent with the ranking of the six-dimensional means.

**Table 3:** Occurrence rates of selected second-level coding items.

First-level dimension	Coding item	Cities	Occurrence rate
Equity protection	Protection against digital exclusion	4	8.3%
Care-needs assessment and regulation	Clear assessment criteria	48	100.0%
Care-needs assessment and regulation	Assessment procedure	48	100.0%
Socialization of family care responsibility	Availability of home-based or visiting services	42	87.5%
Socialization of family care responsibility	Recognition of family or relative caregivers	28	58.3%
Socialization of family care responsibility	Family caregiver training support	17	35.4%
Socialization of family care responsibility	Family care cash or payment support	12	25.0%
Socialization of family care responsibility	Respite care	1	2.1%
Socialization of family care responsibility	Family participation in assessment or care plans	12	25.0%
Socialization of family care responsibility	Home-based or visiting service package	1	2.1%
Payment protection	Account or fund management	45	93.8%
Payment protection	Home-based care payment	7	14.6%
Payment protection	Clear payment proportion	47	97.9%
Digital governance	Online application or assessment	4	8.3%
Digital governance	Age-friendly digital services	1	2.1%
Socialized service provision	Formal care institutions	45	93.8%
Socialized service provision	Diversified service provision	45	93.8%

Occurrence rate, number of cities in which the item appears/48 cities.

**Figure 2:**
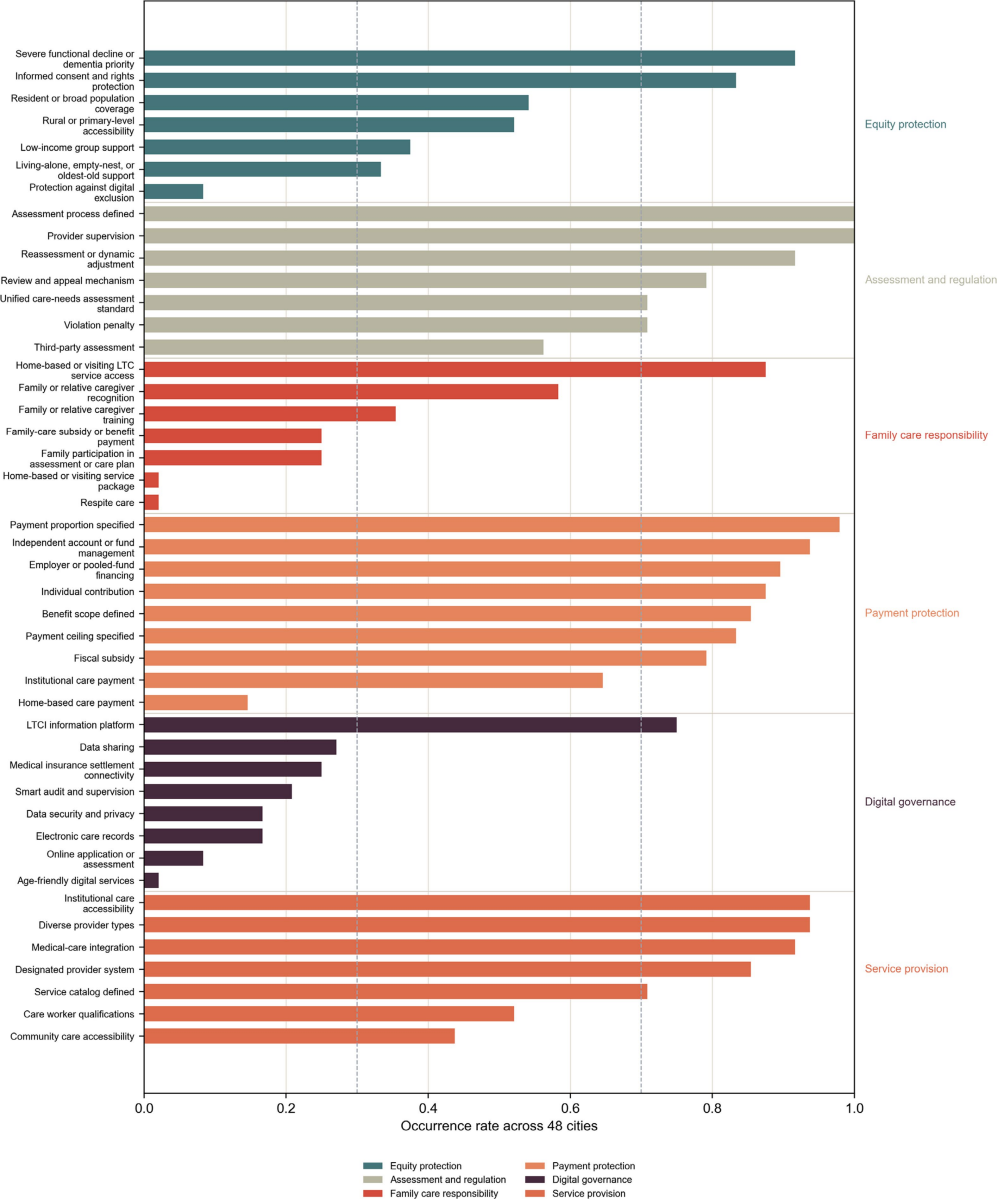
Frequencies of the 45 coding items in the 48 local policy texts.

The socialization of family care responsibility dimension is internally differentiated. Availability of home-based or visiting LTC services appears in 42 cities, and recognition of family or relative caregivers appears in 28 cities, indicating that many local texts retain home-based care settings and relative roles. By contrast, family caregiver training appears in 17 cities, family participation in assessment or care planning and family-care cash or payment support each appear in 12 cities, and respite care and home-based or visiting service packages each appear in only one city. Local policy texts therefore more often recognize home-based care settings, but less often specify caregiver support as operational rules such as training, subsidies, respite care, and participation in care planning. Low-frequency items also concentrate in digital governance and detailed family-support tools, suggesting that local pilot texts still provide insufficient institutional expression for digital procedures, age-friendly application channels, information protection, and detailed caregiver support (Figure 2).

### 4.3 Expression types of city policy texts

**Figure 3:**
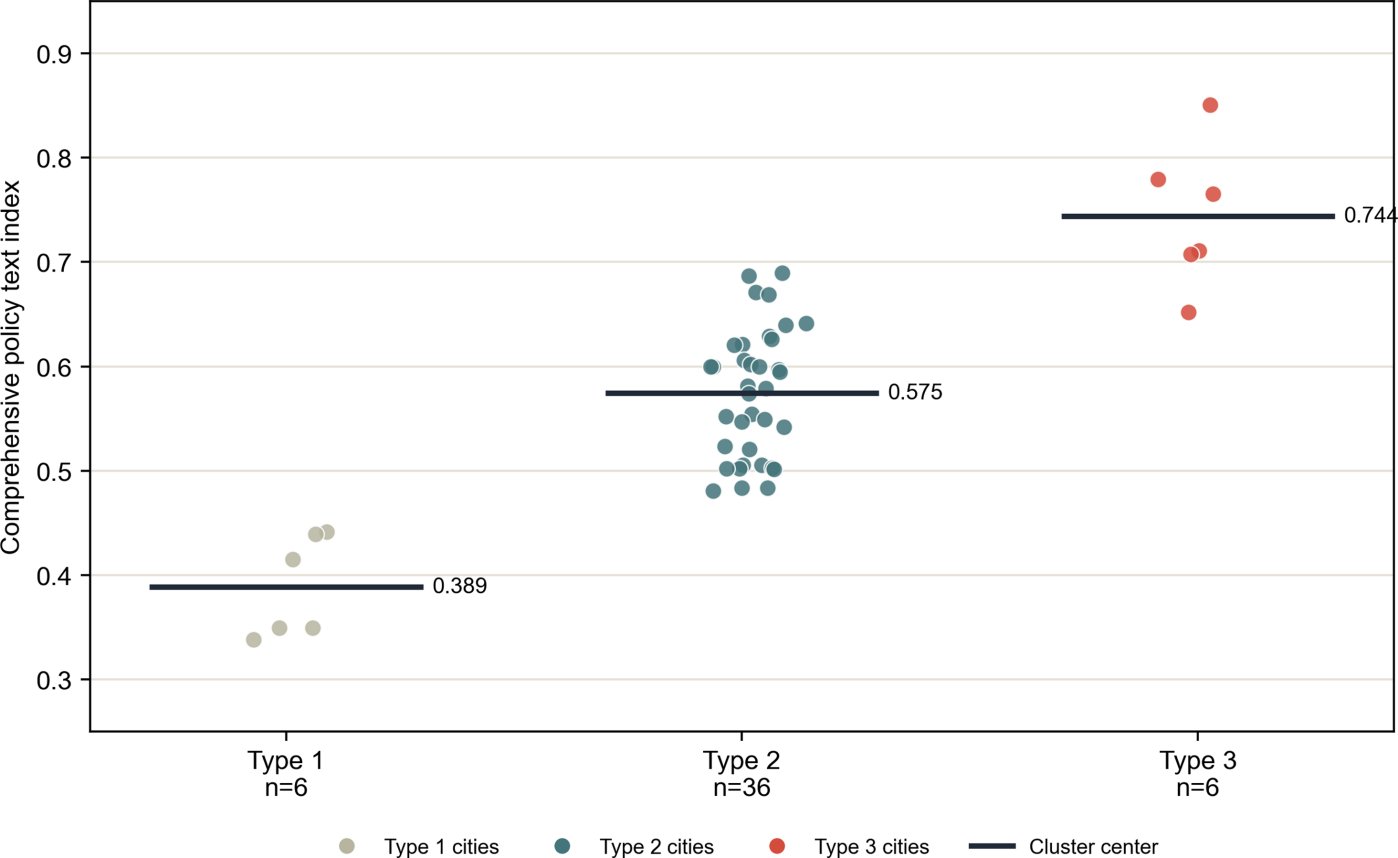
Distribution of the comprehensive policy text index by cluster.

**Figure 4:**
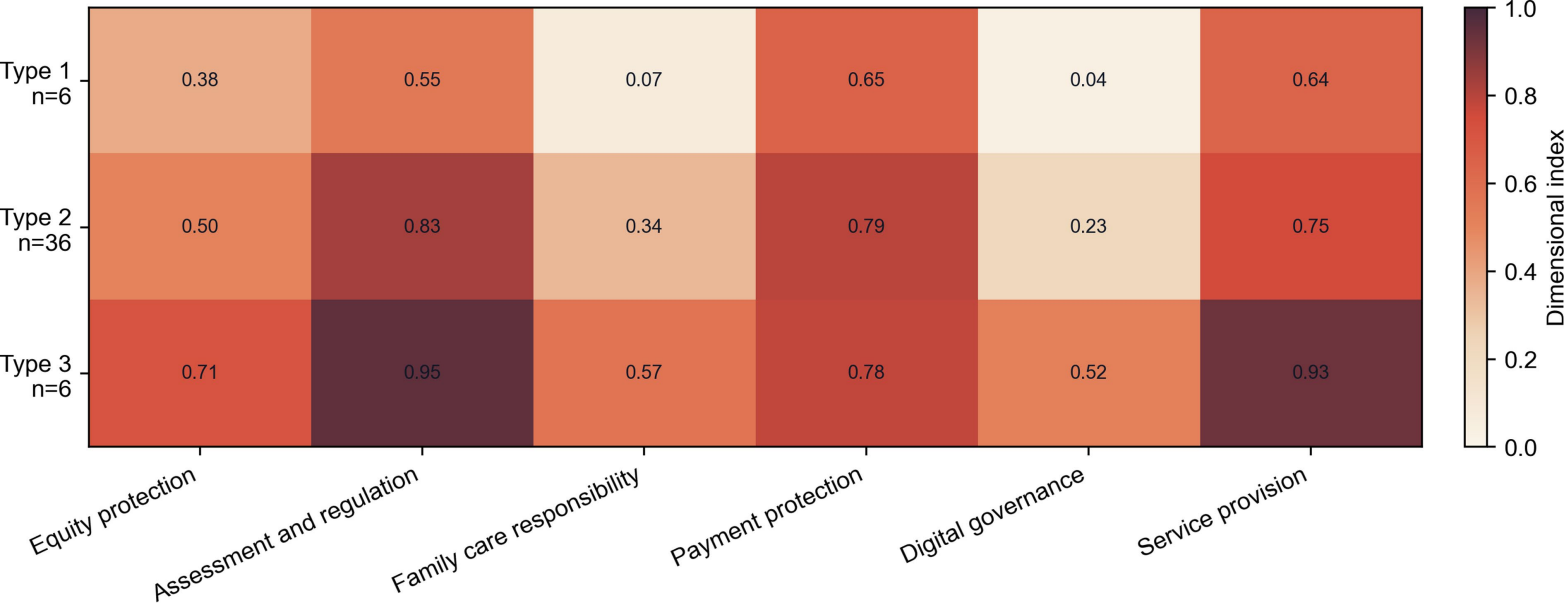
Six-dimensional institutional expression differences across the three pilot-city clusters.

K-means clustering based on the six dimensional indices identifies three local policy text expression types (Figure 3). Table 4 and Figure 4 reports the cluster centers. Type 1 includes six cities and has a comprehensive center value of 0.389. These texts have some payment and service provision rules but weak expression in family care responsibility socialization and digital governance. Type 2 includes 36 cities and has a comprehensive center value of 0.575. This type represents the majority of local pilots, with a relatively clear assessment, payment, and service provision structure but incomplete expression of family support, equity protection, and digital inclusion. Type 3 includes six cities and has a comprehensive center value of 0.744. These texts show stronger expression not only in basic assessment, payment, and service provision, but also in equity protection, family care responsibility socialization, digital governance, and socialized service provision.

**Table 4:** Cluster centers of the three policy text expression types.

Cluster	Cities	Equity protection	Assessment and regulation	Family care responsibility	Payment protection	Digital governance	Service provision	Comprehensive center
Type 1	6	0.381	0.548	0.071	0.648	0.042	0.643	0.389
Type 2	36	0.504	0.833	0.341	0.793	0.226	0.750	0.575
Type 3	6	0.714	0.952	0.571	0.778	0.521	0.929	0.744

The comprehensive center is the equal-weighted average of the six dimensional centers. The cluster labels indicate expression types, not policy quality rankings.

**Figure 5:**
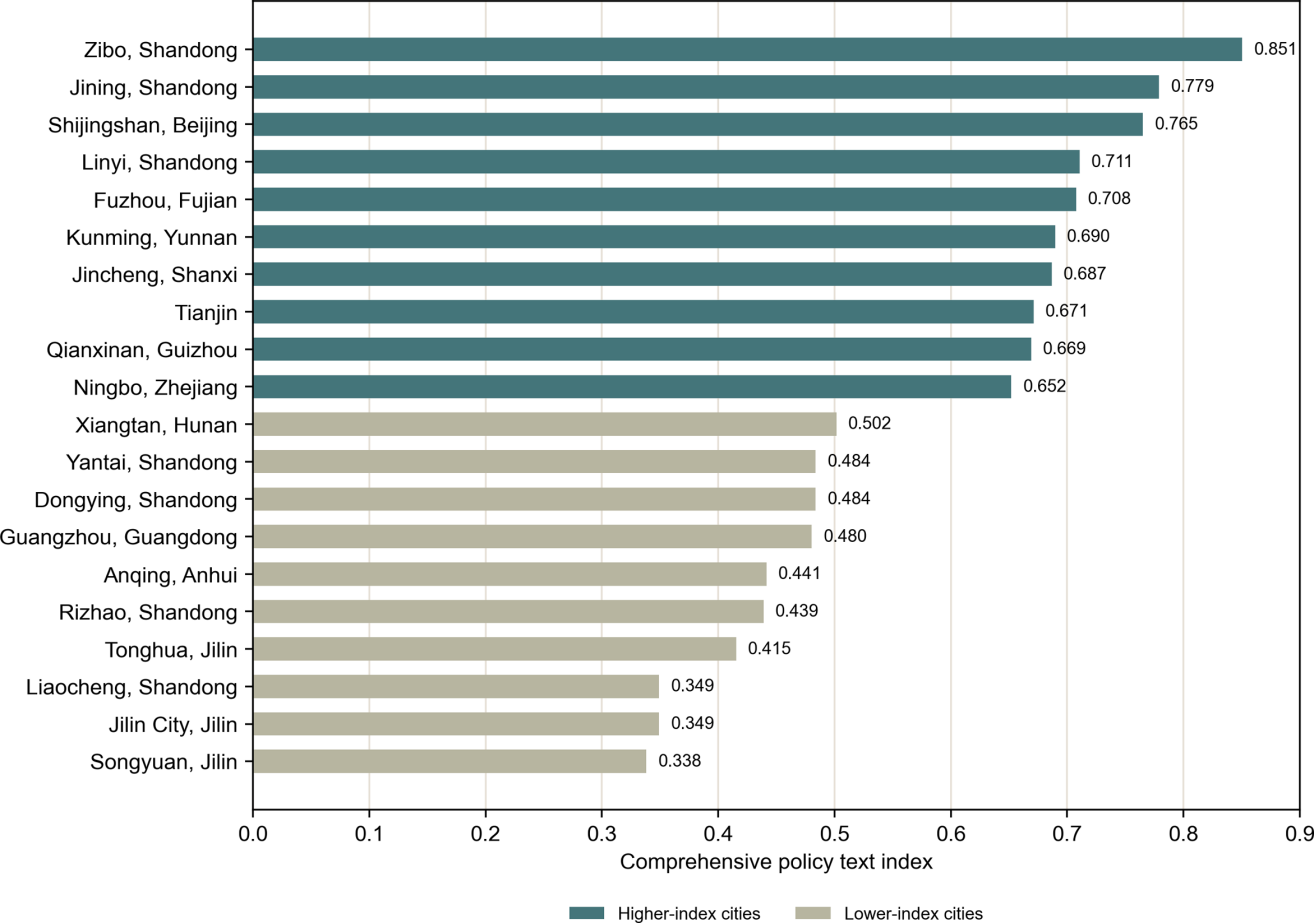
Reference points for the comprehensive policy text index.

The three clusters are not policy quality rankings. They identify combinations of textual expression. The difference between low-, moderate-, and high-expression texts lies mainly in whether a local policy moves beyond the basic framework of assessment, payment, and service provision and writes family support, equity protection, digital accessibility, and service linkage into a more complete institutional structure (Figure 5).

### 4.4 Supplementary reference from the national rollout document

**Figure 6:**
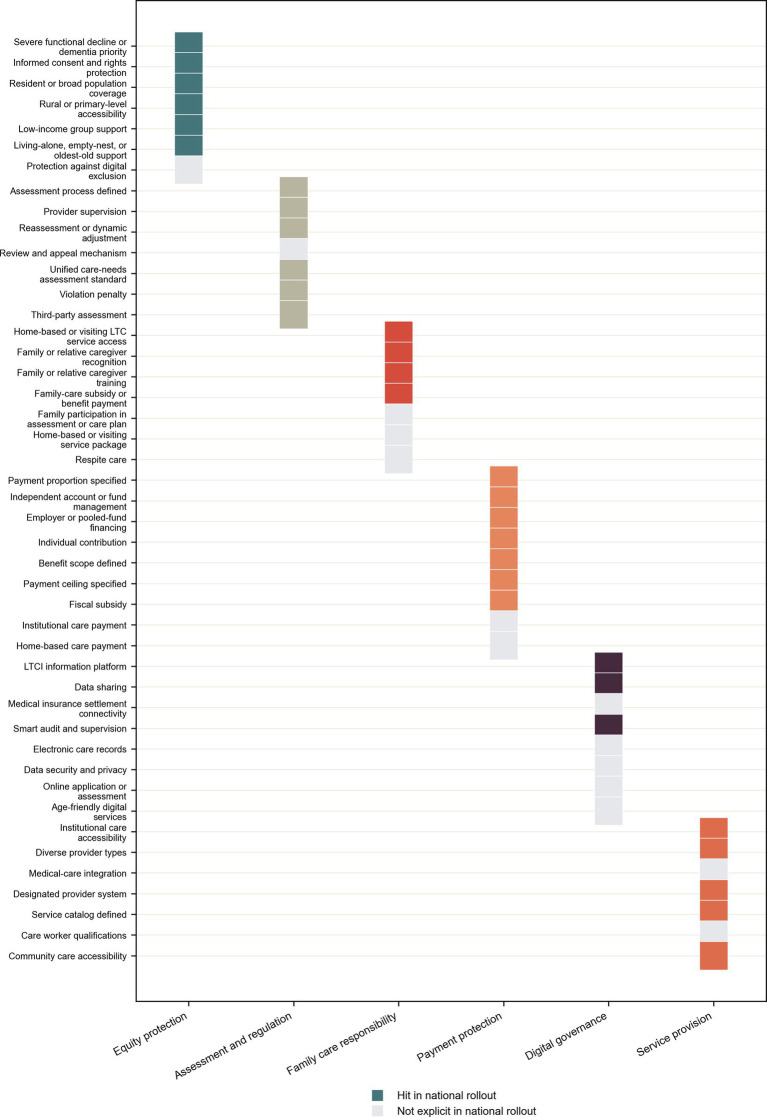
Hit rate of the national rollout document under the same coding framework.

**Figure 7:**
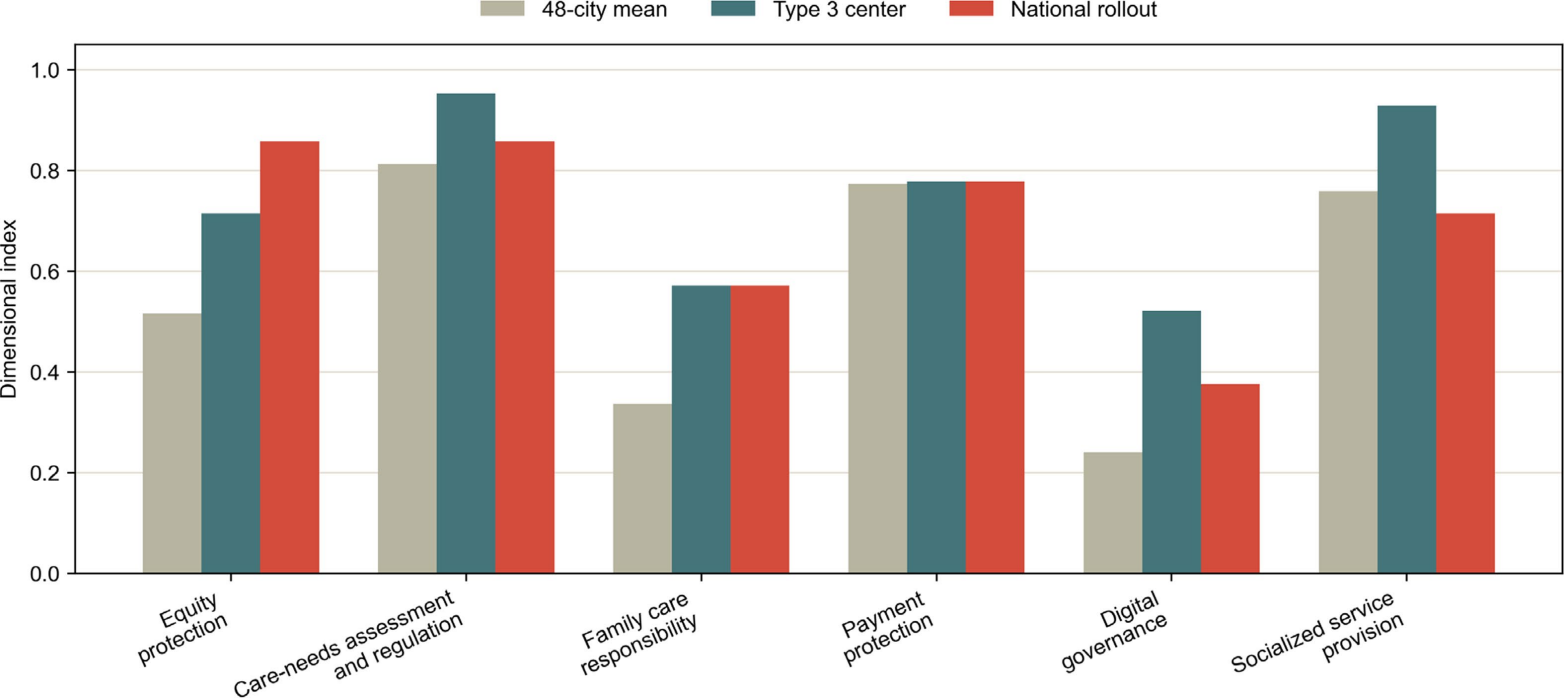
Six-dimensional comparison among the national rollout document, the 48-city mean, and the Type 3 cluster center.

Under the same coding framework, the national rollout document shows both consistency and difference compared with local pilot texts. It hits 31 of the 45 coding items and has a comprehensive index of 0.692, higher than the 48-city mean of 0.573 and lower than the Type 3 center of 0.744. Figure 6 shows the hit positions of the national rollout document in the full 45-item coding framework. At the dimensional level, the national rollout document has an equity protection index of 0.857, higher than the 48-city mean of 0.515 and also higher than the Type 3 center of 0.714. Its socialization of family care responsibility index is 0.571, higher than the local mean of 0.336 and equal to the Type 3 center. This indicates that, compared with the local mean, the national rollout text more clearly writes in elements related to people in difficulty, broad covered groups, family care, and home-based support. The detailed comparison is shown in Table 5 and Figure 7.

**Table 5:** National rollout document compared with local pilot means.

First-level dimension	48-city mean	Type 3 center	National rollout index	National minus 48-city mean
Equity protection	0.515	0.714	0.857	0.342
Care-needs assessment and regulation	0.812	0.952	0.857	0.045
Socialization of family care responsibility	0.336	0.571	0.571	0.235
Payment protection	0.773	0.778	0.778	0.005
Digital governance	0.240	0.521	0.375	0.135
Socialized service provision	0.759	0.929	0.714	−0.045
Comprehensive index	0.573	0.744	0.692	0.120

National minus 48-city mean, national rollout index-48-city mean. All indices range from 0 to 1.

The national rollout document is textually close to the institutional structure already formed in local pilots in assessment, payment, and service provision. The national indices for care-needs assessment and regulation and payment protection are 0.857 and 0.778, respectively, close to the 48-city means. The national index for socialized service provision is 0.714, lower than the 48-city mean of 0.759 and the Type 3 center of 0.929. The digital governance index is 0.375, higher than the local mean of 0.240 but still lower than the Type 3 center of 0.521. The difference between the national text and local pilot texts is therefore not a uniform increase across all dimensions. It is concentrated in equity protection, socialization of family care responsibility, and some digital governance elements.

**Figure 8:**
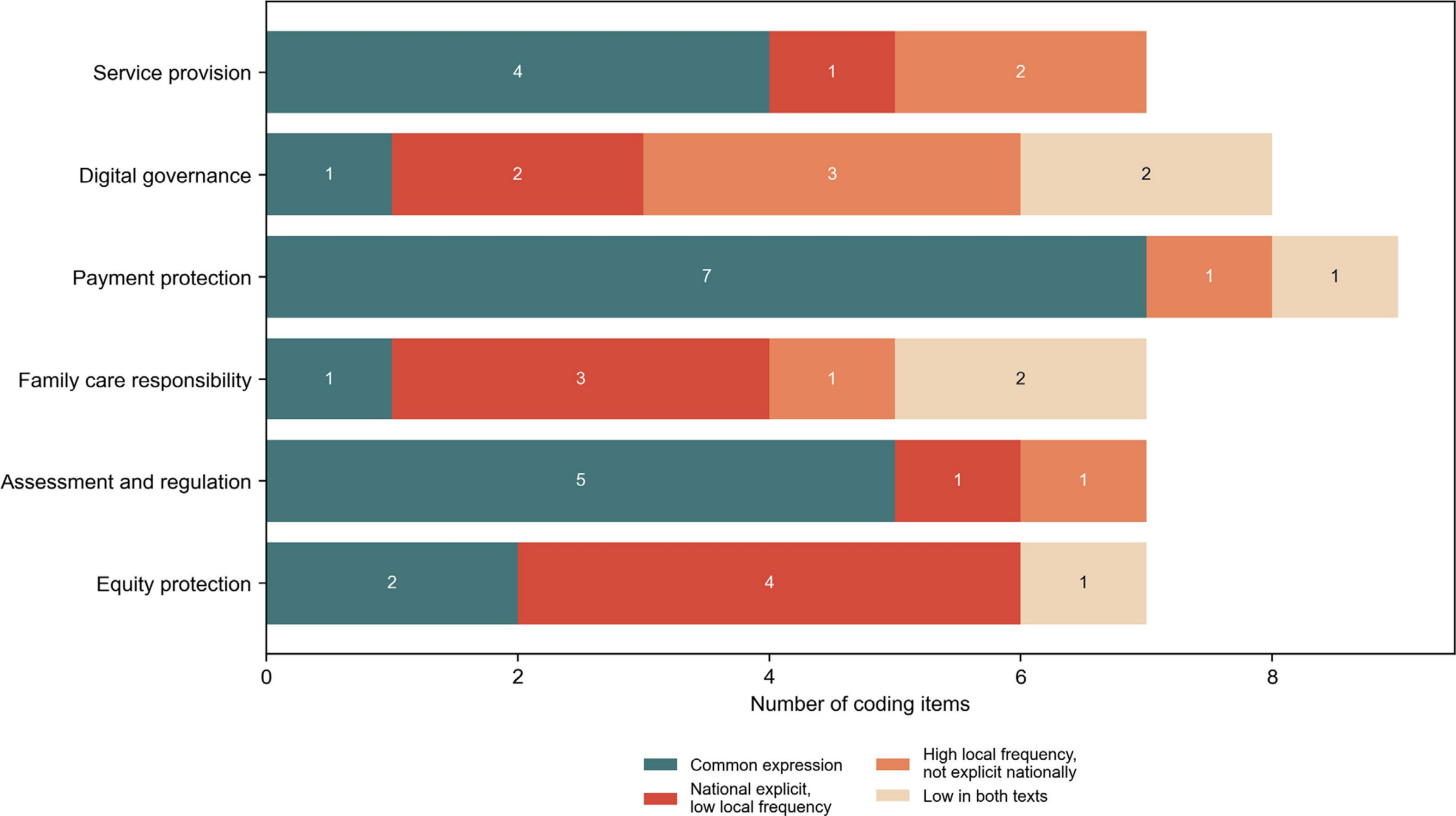
Textual correspondence types between the national rollout document and local pilot institutional elements.

At the coding-item level, the relationship between the national rollout document and local pilot texts can be divided into four categories: common expression, national explicit expression with low local frequency, high local frequency without explicit national expression, and low expression in both texts. Of the 45 coding items, 20 show common expression, 11 are explicit in the national text but low-frequency locally, eight are high-frequency locally but not explicit nationally, and six are low in both (Figure 8). Because the public webpage of the National Healthcare Security Administration contains a deletion note, this section treats the national rollout document only as an open-text reference. The comparison should therefore be read as a supplementary textual comparison, and it does not provide evidence of formal national promotion effects. It cannot support causal claims about diffusion from local pilot experience to national policy, nor can it replace analysis of complete policy texts and policymaking processes.

## 5 Discussion

After long-term care insurance entered the national rollout stage, its institutional meaning exceeded the payment of care costs alone. Local pilot policy texts show that care-needs assessment, payment protection, and socialized service provision form a relatively stable institutional structure, indicating that care for older adults with substantial functional decline is being transformed from an internal family responsibility into a public governance object within the multilevel medical security system. At the same time, socialization of family care responsibility and digital governance remain relatively weak. This suggests that long-term care insurance has begun to assume care pressure that families cannot independently bear, but its expression of family caregiver support, age-friendly digital access, and institutional accessibility for vulnerable groups remains insufficient. The public health meaning of long-term care insurance therefore does not lie only in adding a payment program. It lies in reorganizing care relations among families, communities, service providers, and the state through eligibility determination, payment responsibility, service provision, and regulatory rules, and in translating care ethics such as filial responsibility, care for vulnerable older adults, benevolent rule, and people-centered governance into the governance language of modern medical security.

### 5.1 Confucian care responsibility translation in local pilot policy texts

The filial tradition provides the family ethical starting point for older population care, but Confucian care ethics does not confine care for older adults and vulnerable people within the private household. The Classic of Filial Piety treats filial piety as the root of moral cultivation (31). Mencius extends care from one’s own older adults to the older population of others through the logic of moral extension (32). The Book of Rites imagines an ethical order in which older adults have secure later life and widowed, orphaned, childless, and disabled people are cared for (33). Modern Confucian long-term care studies also point out that filial piety remains an important value resource for understanding long-term care responsibility in Chinese societies, but modern long-term care policy cannot rely simply on family obligation and needs to redistribute responsibility among family care, community support, and formal services (34–36). In this normative chain, filial responsibility points to care responsibility within the family, care for vulnerable older adults points to the response to the vulnerability of older people and people with functional decline, and people-centered governance and benevolent rule require public authority to transform livelihood risks into governable institutional responsibilities.

The most mature institutional expressions in local pilot texts concentrate on care-needs identification, payment responsibility, and formal service organization. This finding indicates that long-term care insurance first constructs an institutional entry point through which care responsibility enters the public system. Care-needs assessment converts a family experience of needing care into policy eligibility. Benefit payment converts long-term cost pressure borne by families into public protection responsibility. Formal service organization connects family care labor with institutions, communities, and professional nursing networks. This institutional chain shows that local policy neither cancels family care responsibility nor shifts care completely to institutions. Instead, it writes care pressure that families cannot independently bear into the public protection system. Filial responsibility thereby receives public support, while care for vulnerable older adults, people-centered governance, and benevolent rule are translated from ethical requirements into public health governance rules that can be identified, paid for, and organized.

This interpretation connects with existing policy-effect research on long-term care insurance. Studies have examined the influence of long-term care insurance on older adults and their families from the perspectives of health status, subjective wellbeing, and benefit design (7, 11, 12), and have analyzed institutional consequences in medical expenditure, service utilization, and hospitalization services (8–10). These studies show that long-term care insurance has changed the resource conditions faced by older adults and families when they encounter care risks. The policy text analysis in this study further suggests that these outcomes do not come from benefit payment alone. They are also related to rules of responsibility identification, cost sharing, and service organization already established in local texts. In other words, the public health meaning of long-term care insurance is reflected not only in health or economic outcomes after implementation, but also in how institutional construction translates family ethical responsibility into public protection responsibility.

Institutions related to family care remain in a transitional form between family ethics and public protection. Local pilot texts have relatively stably established care-needs assessment, benefit payment, and formal service provision rules, and they pay more attention to extending care settings into the family through home-based or visiting services. This result suggests that the modern translation of Confucian care responsibility through long-term care insurance first completes the basic institutional entry point, while the recognition, support, and governance participation of family caregivers show more pronounced differences across local pilots.

### 5.2 Local text-type differences and the degree of responsibility translation

The degree to which filial responsibility is transformed in local pilot texts first depends on whether policy turns the family from a moral care subject into an institutionally supported subject. Home-based or visiting nursing is recognized in most texts, and relative roles also enter care arrangements in some local policies, indicating that long-term care insurance does not remove the family from care relations. Local policies more commonly use home-based services, benefit payment, and links with institutional services to support care pressure that families cannot independently bear. Existing studies show that long-term care insurance changes informal care use, care input by adult children, and formal care utilization (14, 15, 29), and studies on care burden and labor supply also show that policy implementation enters family life arrangements (13, 18). From the policy text perspective, however, institutional support for filial responsibility is already often reflected in services entering the home and cost-pressure sharing, while arrangements that treat family caregivers as direct support objects remain insufficient. The limited expression of caregiver training, family participation in assessment and care planning, respite care, and payment for family care means that family caregivers have not yet generally obtained capacity building, short-term substitute support, or participation in care decision-making, and family ethical responsibility has not yet been fully translated into shared care responsibility supported by public institutions.

This difference in the transformation of filial responsibility is further developed across the three local pilot text types. Low-expression cities have written some payment and service provision rules, but family care responsibility socialization and digital governance are weak, so long-term care insurance appears more as the construction of a basic protection system. In these texts, family care pressure enters the framework of public payment and service provision, but family caregivers remain largely implicit. Moderate-expression cities have a clearer assessment, payment, and service provision structure, yet they have not fully addressed family support, equity protection, and digital inclusion. High-expression cities go further by placing caregiver support, protection for people in difficulty, service accessibility, and digital governance more closely within the policy structure. All three types respond to the same risk of functional decline and care needs. The difference lies in whether local policy connects filial responsibility, care for vulnerable older adults, people-centered governance, and benevolent rule into a more complete institutional chain, moving from family care obligation, to the response to the vulnerability of older people and people with functional decline, and then to the responsibility of public authority for payment, services, and governance.

Care for vulnerable older adults, people-centered governance, and benevolent rule acquire more concrete institutional meanings through these type differences. Care for vulnerable older adults is not only the inclusion of older people as covered populations. It also requires policies to recognize the vulnerability of older adults, people with functional decline, and people in difficulty in access to care. People-centered governance is not only expanded coverage. It also requires that care services can be entered, used, and continuously obtained. Benevolent rule is not only public payment responsibility. It also requires assessment, regulation, data protection, and age-friendly procedures that jointly lower the threshold for vulnerable groups to enter the system. The differentiation of local pilot texts shows that Confucian care ethics can be more fully expressed in modern long-term care insurance only when policy translates these normative requirements into executable rules on covered populations, payment responsibility, family support, service provision, and governance procedures.

This finding also redefines the explanatory premise of existing policy-effect research. Existing studies have shown that long-term care insurance changes the relationship between formal and informal care and affects family care burden and labor supply (13–15, 18, 29). These changes do not occur in homogeneous institutional environments. Cities with more complete textual expression are more likely to leave policy space for family caregiver support, equity protection, and service accessibility. Cities with weaker textual expression remain closer to a basic payment and service provision framework. Long-term care insurance research therefore needs to explain not only household behavior after policy implementation, but also how local pilots arrange care responsibility among families, service providers, communities, and the state at the stage of institutional construction.

### 5.3 Responsibility integration and institutional strengthening in the national rollout document

The supplementary comparison with the national rollout document indicates that the national-level text shows textual continuity with institutional structures commonly observed in local pilots and more clearly expresses family care responsibility and equity protection, which are relatively weaker in local texts. Local pilot texts show that long-term care insurance can write care pressure that families cannot independently bear into the public protection system. The national document further shows that when long-term care insurance enters national rollout, the policy text begins to concentrate more on protection for people in difficulty, broad coverage, home-based care, and family support. Filial responsibility, care for vulnerable older adults, people-centered governance, and benevolent rule are therefore no longer only scattered expressions in local pilot texts. They gain a more unified organizational form in the national institutional text.

The strengthening of family care responsibility socialization in the national rollout document responds to the insufficient support for family caregivers in local pilot texts. Local policies generally recognize home-based or visiting care settings, but less often specify caregiver training, respite care, family participation in care planning, or payment for family care. The national text gives more attention to home-based care, family care, and related support arrangements, suggesting that the national rollout stage gives the family a clearer institutional status in care arrangements. Policy preference and discrete choice experiment studies show that the public has differentiated needs regarding care modes, payment arrangements, and service provision (7, 53), and care needs among people with dementia and older adults with substantial functional decline require more detailed institutional responses (54, 55). From the perspective of Confucian care ethics, the key is not to weaken family responsibility, but to provide more stable public support for families to fulfill care responsibility.

Stronger equity protection reflects the national rollout stage’s institutional response to care for vulnerable older adults and people-centered governance. In local pilots, equity protection is weaker than assessment, payment, and service provision, indicating that although care risks have entered the public protection system, people in difficulty, special groups, and differences in service accessibility still need clearer rules. The national rollout document places more emphasis on people in difficulty and broad coverage, providing a clearer equity orientation for the transition from pilot schemes to a national system. People-centered governance here is not an abstract value declaration. It is expressed through rules on coverage, benefit linkage, service access, and protection of vulnerable groups. Long-term care insurance can move older adults with care needs and their families into the system only if these links are further specified.

The national rollout document still leaves room for further improvement, especially in detailed rules on digital governance and family caregiver support. National rollout requires more intensive information management, but whether older adults, people with substantial functional decline, and groups with low digital literacy can complete application, assessment, service selection, and information confirmation directly affects institutional accessibility. Local pilot texts have already shown that age-friendly digital services, protection against digital exclusion, and data protection are weak links. The national rollout stage also needs to write offline alternatives, community assistance, privacy protection, and data security into more specific implementation rules. In modern public health governance, benevolent rule is expressed not only by expanding institutional supply, but also by designing rules that lower the entry threshold for vulnerable groups.

### 5.4 Limitations

The evidence boundary of this study comes from policy text analysis itself. Policy texts show whether institutional elements are explicitly written into rules, but they do not directly represent actual service supply capacity, fund operation quality, beneficiary experience, or implementation effects. The findings on family care responsibility socialization, digital governance, and equity protection are therefore limited to textual expression. The Confucian interpretation also remains at the level of discussion. It can explain how local policy texts use covered populations, payment responsibility, service provision, and governance rules to carry normative resources such as filial responsibility, care for vulnerable older adults, benevolent rule, and people-centered governance. It cannot infer whether policymakers directly used Confucian culture as a motive, nor can it prove a causal influence of Confucian culture on policy formation. The contribution of this interpretation is to read institutional expressions through a Confucian care ethics lens rather than to reconstruct policymaker intentions.

The sample and reference text also affect interpretation. This study collected core policy texts according to the 49-city pilot framework and included 48 cities in the main analysis. Shihezi was excluded because no core implementation text meeting the inclusion criteria was available. The national rollout document comes from the public webpage of the National Healthcare Security Administration, and that webpage contains a deletion notice. It is therefore used only as an open-text reference, without representing a complete national policymaking process. The correspondence between the national rollout document and local pilot texts can show common elements, different elements, and strengthening directions under the same coding framework. Policy diffusion research indicates that diffusion may involve learning, imitation, competition, and coercion (56, 57), but this study does not use policymaking-process materials, departmental interviews, or internal documents. It cannot determine whether the relevant expressions in the national text come from the absorption of local pilot experience. This boundary also clarifies the study’s contribution. By separating textual institutional expression from implementation effects, the analysis provides a reproducible baseline for identifying which aspects of care responsibility allocation have already been written into long-term care insurance rules and which require later empirical evaluation. Future research can combine policy text analysis with service utilization data, fund operation data, caregiver interviews, and policymaking-process materials to examine relationships among text expression, implementation processes, and care outcomes.

## 6 Conclusion

This study analyzes core policy texts from 48 long-term care insurance pilot cities by combining public health governance with a Confucian interpretive perspective. The findings show that the institutional meaning of long-term care insurance is not limited to payment of care costs, health improvement, or changes in service utilization. It also lies in how the system converts care for older adults with substantial functional decline from an internal family responsibility into an institutional arrangement within public protection. Through rules on covered populations, care-needs assessment, benefit payment, service provision, and regulation, local policy texts reorganize care responsibility among families, markets, communities, and the state, allowing filial responsibility, care for vulnerable older adults, benevolent rule, and people-centered governance to acquire modern institutional expression. The supplementary comparison with the national rollout document further shows that the national rollout stage gives clearer expression to protection for people in difficulty and family care support, with strengthening in equity protection and socialization of family care responsibility. Digital governance, detailed caregiver support, and home-based service specification still require further improvement. Long-term care insurance has become an important institutional text in China’s multilevel medical security system, linking family ethical responsibility, public protection responsibility, and modern care governance. If later policy further strengthens caregiver support, equity protection, and age-friendly digital governance, long-term care insurance will be better able to translate traditional care ethics into public health protection that is accessible, payable, and regulable.

## Data Availability

The original contributions presented in the study are included in the article/Supplementary material, further inquiries can be directed to the corresponding author.
