# Supplementary material for: Institutionalizing family care responsibility in China’s multilevel medical security system: evidence from long-term care insurance policy texts through a Confucian care ethics lens
Source: Front Public Health. 2026 Jul 31;14:1918294. doi: 10.3389/fpubh.2026.1918294 (PMC13472915; doi:10.3389/fpubh.2026.1918294)
Supplement: Supplementary file 1 [file Table_1.DOCX]

**Supplementary Table S1. Coding Framework Sources and Judgment Rules**

| **First-level dimension** | **Core meaning** | **Second-level coding item** | **Coding source** | **Rule for coding as 1** | **Non-codable / coded as 0** |
| --- | --- | --- | --- | --- | --- |
| Equity protection | Covered populations and protection for vulnerable groups | Resident or broad population coverage | A National pilot institutional design; B Policy evaluation element | The text explicitly states that insured or covered groups include residents, employees, retirees, urban and rural residents, flexible workers, or proposes expansion from specific groups to broader covered populations. | Only broad terms such as eligible persons or insured persons are used, without specifying the scope of covered groups or an expansion direction; general statements in news reports or policy interpretations are not coded. |
| Equity protection | Covered populations and protection for vulnerable groups | Low-income group support | A National pilot institutional design; B Policy evaluation element | The text explicitly includes contribution subsidies, benefit linkage, priority protection, or special support for groups such as minimum-living-standard recipients, extremely poor people, people in difficulty, or people at risk of returning to poverty. | Only goal-oriented language such as reducing burden or improving protection is used, without pointing to specific disadvantaged groups or support rules. |
| Equity protection | Covered populations and protection for vulnerable groups | Rural or primary-level accessibility | A National pilot institutional design; B Policy evaluation element | The text explicitly refers to rural residents, primary-level services, primary-level administration, primary-level service networks, township-level arrangements, or community accessibility. | The policy is only stated as a citywide rule, without rural, primary-level, township, community accessibility, or service-downward arrangements. |
| Equity protection | Covered populations and protection for vulnerable groups | Priority for severe functional decline or dementia | A National pilot institutional design; C Long-term care responsibility relationship | The text explicitly treats people with severe functional decline, long-term functional decline, dementia, or high-intensity care needs as initial, priority, key, or eligible protection groups. | The text only refers generally to older adults, people with functional decline, or care recipients, without indicating severe functional decline, dementia, or priority protection. |
| Equity protection | Covered populations and protection for vulnerable groups | Support for living-alone, empty-nest, or oldest-old groups | B Policy evaluation element; C Family care responsibility relationship | The text explicitly identifies oldest-old, living-alone, empty-nest, childless-care, or specially disadvantaged older adults and sets service priority, benefit linkage, or administrative support. | The text only mentions older adults or older adults with functional decline, without institutional arrangements for living-alone, empty-nest, oldest-old, or other vulnerable situations. |
| Equity protection | Covered populations and protection for vulnerable groups | Protection against digital exclusion | B Policy evaluation element; C Family care responsibility relationship | The text explicitly sets offline handling, proxy assistance, community assistance, alternatives for people without smart devices, or proposes preventing a digital divide or digital exclusion. | The text only builds an online platform, online application, or information system, without non-digital alternatives or digital exclusion protection. |
| Equity protection | Covered populations and protection for vulnerable groups | Informed consent and rights protection | A National pilot institutional design; B Policy evaluation element | The text explicitly includes informed consent, notification of results, complaints and reports, rights remedies, information disclosure, personal rights protection, or dispute handling. | The text only states standardized management or improved service quality, without specific rights-protection rules for insured persons, service users, or families. |
| Care-needs assessment and regulation | Eligibility determination and service constraints | Unified care-needs assessment standard | A National pilot institutional design; B Policy evaluation element | The text explicitly adopts a unified care-needs assessment standard, assessment scale, assessment indicators, or a local unified assessment method. | The text only states that benefits are enjoyed after assessment, without standards, scales, grades, or a unified method. |
| Care-needs assessment and regulation | Eligibility determination and service constraints | Third-party assessment | A National pilot institutional design; B Policy evaluation element | The text explicitly assigns assessment to a third-party organization, professional assessment agency, entrusted assessment organization, or independent assessment force. | Assessment is only conducted by medical security agencies, administrative agencies, or service providers internally, without third-party or independent assessment. |
| Care-needs assessment and regulation | Eligibility determination and service constraints | Assessment process defined | A National pilot institutional design; B Policy evaluation element | The text explicitly states process steps such as application, acceptance, material review, on-site assessment, result confirmation, public notice, or benefit initiation. | The text only mentions applying for assessment or passing assessment, without verifiable process steps. |
| Care-needs assessment and regulation | Eligibility determination and service constraints | Reassessment and dynamic adjustment | A National pilot institutional design; B Policy evaluation element | The text explicitly provides regular reassessment, dynamic adjustment, benefit eligibility review, reassessment after changes in care-needs grade, or exit mechanisms. | The text only provides initial assessment or eligibility determination, without reassessment, dynamic adjustment, or exit rules. |
| Care-needs assessment and regulation | Eligibility determination and service constraints | Review and appeal mechanism | A National pilot institutional design; B Policy evaluation element | The text explicitly provides objection to assessment results, review application, reassessment, appeal, or dispute handling procedures. | The text only provides consultation numbers, policy explanation, or general complaint channels, without pointing to review of assessment results. |
| Care-needs assessment and regulation | Eligibility determination and service constraints | Provider supervision | A National pilot institutional design; B Policy evaluation element | The text explicitly provides designated-provider supervision, agreement management, service quality inspection, expense review, performance assessment, or exit management. | The text only states strengthening management or improving service levels, without supervisory objects, procedures, or tools. |
| Care-needs assessment and regulation | Eligibility determination and service constraints | Violation penalty | A National pilot institutional design; B Policy evaluation element | The text explicitly provides penalties, recovery, suspension, or cancellation of eligibility for insurance fraud, false assessment, irregular charges, excessive services, or fund misappropriation. | The text only prohibits violations or standardizes behavior, without consequences such as penalty, recovery, suspension, or cancellation of eligibility. |
| Socialization of family care responsibility | Family care settings and support for family caregivers | Availability of home-based or visiting LTC services | A National pilot institutional design; C Family care responsibility relationship | The text explicitly includes home-based nursing, visiting nursing, home-based care, visiting services, or home-based care benefits in long-term care insurance service modes or benefit scope. Illustrative positive evidence from coded policy texts: Shanghai defined community home care as services provided to home-dwelling insured persons through door-to-door or community care by elder-care institutions, nursing stations, community health service centers, or nursing homes. | General expressions such as reducing family burden, supporting family-based old-age care, giving play to the family role, or advocating home-based ageing are not coded by themselves unless they are accompanied by explicit home-based or visiting service arrangements under long-term care insurance. Boundary example: a general statement supporting home-based ageing or family-based elder care was not coded unless the text specified an LTCI service mode, service list, or benefit rule. |
| Socialization of family care responsibility | Family care settings and support for family caregivers | Recognition of family or relative caregivers | B Policy evaluation element; C Family care responsibility relationship | The text explicitly treats family members, relatives, family caregivers, or household caregivers as care providers, service participants, assistants, service-confirmation actors, management, training, or support objects, or explicitly supports family members' participation in long-term care. Illustrative positive evidence from coded policy texts: Beijing Shijingshan specified that home care may include care services provided by institutional care workers or by the disabled person's relatives, described as domestic caregivers. | General expressions such as reducing family burden, supporting family-based old-age care, giving play to the family role, or supporting families are not coded by themselves unless the text specifies a concrete role of family members, relatives, family caregivers, or household caregivers in care, assistance, confirmation, management, training, subsidy, or support. Boundary example: family members appearing only as background actors, contacts, or general moral supporters were not coded without a concrete role in care provision, confirmation, training, subsidy, or management. |
| Socialization of family care responsibility | Family care settings and support for family caregivers | Family or relative caregiver training support | C Family care responsibility relationship | The text explicitly provides care training, nursing guidance, skills training, or care-capacity building for family members, relatives, or family caregivers. Illustrative positive evidence from coded policy texts: Kunming included home care provided by family members who had received caregiver training as one long-term care insurance benefit mode. | Training is only provided for institutional care workers, service personnel, or assessors, not for family caregivers. Boundary example: training for professional care workers, assessors, managers, or designated institutional staff was not coded as family caregiver training. |
| Socialization of family care responsibility | Family care settings and support for family caregivers | Cash subsidy or benefit payment for family or relative care | B Policy evaluation element; C Family care responsibility relationship | The text explicitly pays subsidies, cash benefits, nursing allowances, or service fees to family caregivers, relative caregivers, or home-based care providers. Illustrative positive evidence from coded policy texts: Beijing Shijingshan set a payment standard for eligible home-care services provided by relatives, with the fund paying a specified share of the hourly service price. | Home-based service fees are paid only to institutions or service personnel, not to family or relative caregivers. Boundary example: home-care payment made only to designated institutions or professional personnel was not coded as family or relative care payment. |
| Socialization of family care responsibility | Family care settings and support for family caregivers | Respite care | C Family care responsibility relationship | The text explicitly includes respite care, short-term substitute care, temporary day or residential care, or substitute services designed to relieve family caregivers. Illustrative positive evidence from coded policy texts: Binzhou's dementia-related long-term care insurance application form listed respite care as an application category together with long-term care and day care. | Short-term hospitalization, temporary services, or institutional care are mentioned without indicating respite or substitute care for family caregivers. Boundary example: temporary hospitalization, short-term institutional care, or general day care was not coded unless the text indicated a respite or substitute-care function for family caregivers. |
| Socialization of family care responsibility | Family care settings and support for family caregivers | Family participation in assessment, care planning, or service choice | A National pilot institutional design; C Family care responsibility relationship | The text explicitly provides family participation in assessment, care-plan formulation, service item selection, service confirmation, informed signature, or care decision-making. Illustrative positive evidence from coded policy texts: Jinan required designated service institutions to formulate care service plans through consultation with the insured person or family members, with the plan specifying service items, frequency, time, and providers. | Family members appear only as proxy applicants, material submitters, or contacts, without participation in assessment, planning, or service decisions. Boundary example: family members acting only as proxy applicants, contacts, or material submitters were not coded without participation in assessment, planning, service choice, service confirmation, or informed signature. |
| Socialization of family care responsibility | Family care settings and support for family caregivers | Home-based or visiting service package | A National pilot institutional design; C Family care responsibility relationship | The text explicitly lists home-based or visiting service packages, service lists, service combinations, or specific home-based nursing items. Illustrative positive evidence from coded policy texts: Zibo required home-care service packages and payment ceilings, with the package determined through consultation between the insured person and the designated care institution. | The text only says that home-based nursing or visiting services are provided, without service packages, lists, or specific items. Boundary example: a policy that merely allowed home-based or visiting care was not coded as a service package unless it listed packages, service lists, specific items, or package-based payment limits. |
| Payment protection | Financing, fund management, and benefit payment | Account or fund management | A National pilot institutional design; B Policy evaluation element | The text explicitly provides long-term care insurance funds, special account management, separate accounting, fund budgeting, fund revenue and expenditure, or independent account management. | The text only mentions financing or benefit payment, without fund accounts, accounting, or management rules. |
| Payment protection | Financing, fund management, and benefit payment | Fiscal subsidy | A National pilot institutional design; B Policy evaluation element | The text explicitly provides fiscal subsidies, government subsidies, public finance assistance, or government responsibility for part of financing. | The text only states diversified financing, social fundraising, or fund raising, without specifying fiscal or government funding responsibility. |
| Payment protection | Financing, fund management, and benefit payment | Individual contribution | A National pilot institutional design; B Policy evaluation element | The text explicitly provides individual contribution, insured-person contribution, resident contribution, or retiree individual contribution. | Only employer, pooled fund, or fiscal financing is provided, without individual contribution. |
| Payment protection | Financing, fund management, and benefit payment | Employer or pooled-fund financing | A National pilot institutional design; B Policy evaluation element | The text explicitly provides employer contribution, employing-unit contribution, transfer from employee medical insurance pooled funds, fund adjustment, or pooled-fund financing. | The text only states diversified financing sources, without specifying employer or pooled-fund responsibility. |
| Payment protection | Financing, fund management, and benefit payment | Home-based care payment | A National pilot institutional design; B Policy evaluation element; C Family care responsibility relationship | The text explicitly provides fund payment proportion, payment standard, ceiling, or benefit rule for home-based care, visiting services, or home-based nursing. | Home-based service accessibility is provided, but payment standards, payment proportions, or benefit payment are not specified. |
| Payment protection | Financing, fund management, and benefit payment | Institutional care payment | A National pilot institutional design; B Policy evaluation element | The text explicitly provides fund payment proportion, payment standard, ceiling, or benefit rule for institutional nursing, institutional residence, or designated-provider services. | Institutions appear only as service providers, without institutional care payment benefits. |
| Payment protection | Financing, fund management, and benefit payment | Payment proportion specified | A National pilot institutional design; B Policy evaluation element | The text explicitly gives fund payment proportions, reimbursement proportions, individual out-of-pocket proportions, or proportions corresponding to different service modes. | The text only says payment according to rules, reasonable payment, or fund payment of part of expenses, without a proportional rule. |
| Payment protection | Financing, fund management, and benefit payment | Payment ceiling specified | A National pilot institutional design; B Policy evaluation element | The text explicitly provides daily, monthly, annual, bed-day, item, or capped payment limits. | The text only mentions cost control, reasonable payment, or benefit standards, without specified ceilings or caps. |
| Payment protection | Financing, fund management, and benefit payment | Benefit scope defined | A National pilot institutional design; B Policy evaluation element | The text explicitly defines payable service scope, benefit items, items not paid by the fund, or benefit boundaries. | The text only says that long-term care insurance benefits are enjoyed, without defining benefit scope or exclusions. |
| Digital governance | Administration, regulation, and data-based governance | LTCI information platform | A National pilot institutional design; B Policy evaluation element | The text explicitly establishes or uses a long-term care insurance information platform, business system, administrative system, or information-management platform. | The text only generally promotes informatization or information management, without referring to an LTCI platform or business system. |
| Digital governance | Administration, regulation, and data-based governance | Medical security settlement connectivity | A National pilot institutional design; B Policy evaluation element | The text explicitly connects long-term care insurance with medical security settlement systems, medical security administration, medical security payment, or medical security information systems. | The text only mentions ordinary financial settlement, institutional settlement, or service settlement, without connection to medical security systems or administration. |
| Digital governance | Administration, regulation, and data-based governance | Online application or assessment | B Policy evaluation element | The text explicitly allows or requires online application, online acceptance, online assessment appointment, online query of assessment results, or online processing. | Policy information is only published on a government website, or an information platform is built without online application or assessment. |
| Digital governance | Administration, regulation, and data-based governance | Data sharing | A National pilot institutional design; B Policy evaluation element | The text explicitly provides data sharing, information connectivity, or result mutual recognition among departments such as medical security, civil affairs, health, human resources and social security, disability federations, or tax authorities. | The text only records or reports data within a department, without cross-department sharing. |
| Digital governance | Administration, regulation, and data-based governance | Electronic care records | B Policy evaluation element | The text explicitly uses electronic care records, electronic service records, electronic sign-in, electronic care logs, or online service records. | Only paper-based nursing records, service records, or archive management are required, without electronic records. |
| Digital governance | Administration, regulation, and data-based governance | Smart audit and supervision | B Policy evaluation element | The text explicitly uses smart audit, smart supervision, video monitoring, location monitoring, data monitoring, big-data risk control, or automatic screening of abnormal expenses. | Only manual review, on-site inspection, or general supervision is provided, without intelligent or data-based regulation. |
| Digital governance | Administration, regulation, and data-based governance | Data security and privacy | A National pilot institutional design; B Policy evaluation element | The text explicitly provides personal information protection, data security, privacy protection, information security responsibility, or boundaries for data use. | The text only generally requires confidentiality, without data, personal information, privacy, or information security rules. |
| Digital governance | Administration, regulation, and data-based governance | Age-friendly digital services | B Policy evaluation element; C Family care responsibility relationship | The text explicitly proposes age-friendly online services, older-adult-friendly interfaces, digital assistance, coordination between offline alternatives and online services, or digital service optimization for older adults. | The text only provides online services or an information platform, without age-friendly, older-adult-friendly, or assisted-use arrangements. |
| Socialized service provision | Professional service networks and service responsibility | Institutional care accessibility | A National pilot institutional design; B Policy evaluation element; C Long-term care responsibility relationship | The text explicitly includes eldercare institutions, nursing institutions, medical institutions, designated institutions, or other institutional care within service modes or protection scope. | Institutions are mentioned only for management or qualification purposes, without institutional care service accessibility. |
| Socialized service provision | Professional service networks and service responsibility | Community care accessibility | A National pilot institutional design; B Policy evaluation element; C Long-term care responsibility relationship | The text explicitly includes community care, community nursing stations, community service centers, day care, or community visiting services in service provision. | Community appears only as an administrative area or administrative site, without community care services. |
| Socialized service provision | Professional service networks and service responsibility | Designated provider system | A National pilot institutional design; B Policy evaluation element | The text explicitly provides designated-provider admission, agreement management, designated service lists, designated nursing institutions, or designated-provider management. | Only names of service institutions or service sites appear, without designated admission or agreement management. |
| Socialized service provision | Professional service networks and service responsibility | Diverse provider types | A National pilot institutional design; B Policy evaluation element; C Long-term care responsibility relationship | The text explicitly provides multiple types of providers, such as medical institutions, eldercare institutions, nursing institutions, community organizations, social forces, or market actors jointly participating. | The text only contains a single provider type or generally refers to service institutions, without diverse provision. |
| Socialized service provision | Professional service networks and service responsibility | Service catalog defined | A National pilot institutional design; B Policy evaluation element | The text explicitly lists a long-term care service catalog, service item list, nursing service package, service content, or payable items. | The text only says that nursing services, daily-life care, or medical nursing are provided, without catalogs, lists, or item boundaries. |
| Socialized service provision | Professional service networks and service responsibility | Care worker qualifications | A National pilot institutional design; B Policy evaluation element; C Long-term care responsibility relationship | The text explicitly provides qualifications, training, certification, registration, or capability requirements for nursing workers, care workers, service personnel, or assessors. | Only institutional qualification or institutional admission is provided, without capability or qualification requirements for specific care personnel. |
| Socialized service provision | Professional service networks and service responsibility | Medical-care integration | A National pilot institutional design; B Policy evaluation element; C Long-term care responsibility relationship | The text explicitly connects long-term care insurance with medical services, eldercare services, rehabilitation nursing, family doctors, or integrated medical and eldercare services. | Medical care and eldercare are only mentioned separately, without a linkage mechanism, service coordination, or responsibility connection. |

**Note:** This table lists the sources and judgment rules for the 45 second-level coding items. Coding sources are defined as follows: A = institutional design in the national pilot documents; B = policy elements repeatedly used in previous long-term care insurance policy evaluation studies; C = responsibility relationships shown to be important in family care and long-term care studies. Confucian cultural translation is used only as an interpretive framework in the Discussion and is not treated as a coding source. Coding followed a conservative principle: an item was coded as 1 only when the policy text explicitly wrote rules on covered objects, procedures, payment, services, regulation, or support. General policy goals, news interpretations, policy publicity materials, single payment notices, and explanatory pages without institutional rules were not used as coding evidence. General expressions such as reducing family burden, supporting family-based old-age care, or giving play to the family role were not by themselves inferred as specific coding items. When such expressions appeared together with institutional arrangements such as home-based care, visiting services, family-member participation, family confirmation, family caregiver training, or caregiver subsidy, coding followed the specific rule for the corresponding item. Illustrative positive examples in the family-care responsibility dimension are paraphrased from coded local policy texts and are used only to clarify coding judgments.
